# The Effectiveness of Yoga for Irritable Bowel Syndrome: A Systematic Review

**DOI:** 10.1002/cph4.70061

**Published:** 2025-10-18

**Authors:** Francesco Pavan, Sunil Singh Yadav, Andrea Costantino, Alessandra Dell'Era, Monic Mastroianni, Massimiliano Buoli

**Affiliations:** ^1^ Department of Pathophysiology and Transplantation University of Milan Milan Italy; ^2^ Faculty of Naturopathy and Yogic Sciences Shree Guru Govind Tricentenary, SGT University Gurugram Haryana India; ^3^ Gastroenterology and Endoscopy Unit Foundation IRCCS Ca' Granda Ospedale Maggiore Policlinico Milan Italy; ^4^ Yogamilan Milan Italy; ^5^ Department of Neurosciences and Mental Health Fondazione IRCCS Ca' Granda, Ospedale Maggiore Policlinico Milan Italy

**Keywords:** complementary therapy, irritable bowel syndrome, systematic review, yoga

## Abstract

**Background:**

Irritable Bowel Syndrome (IBS) is a common functional gastrointestinal disorder affecting 5%–10% of the global population and is characterized by recurrent abdominal pain and dysregulation of bowel movements. Many patients do not benefit from full remission of symptoms by conventional treatments. Recently, yoga has demonstrated its potential benefits in various medical conditions including IBS, as a result of its effect on the gut‐brain axis. This systematic review evaluates the effectiveness of yoga for the management of IBS.

**Methods:**

We conducted a comprehensive literature search in PubMed and Scopus until March 2025 to identify studies on yogic posture, pranayama, and meditation in IBS. After excluding reviews, case reports, and studies not meeting age or design criteria, 10 studies were included. These were evaluated for methodological quality and classified as randomized controlled trials (RCTs) or non‐controlled trials. We analyzed outcomes related to gastrointestinal symptoms, quality of life, and psychological symptoms and examined the type of yoga administered.

**Results:**

Yoga interventions led to amelioration of gastrointestinal symptoms, decreased anxiety and depression, and enhanced quality of life compared to usual care or wait‐list controls. Of note, the studies that fixed the change in the IBS Symptom Severity Scale scores as the primary outcome showed a moderate‐large effect of yoga on the improvement of symptoms compared to controls (Cohen's *d* range: 0.37–3.60).

**Conclusion:**

These preliminary findings suggest that yoga could favorably influence both the physical and psychological aspects of IBS. However, high‐quality larger sample studies are needed to confirm the findings reported in the review.

## Graphical abstract

Evidence from 10 studies suggests yoga improves IBS symptoms, stress/anxiety, and QOL; study quality was moderate, supporting the need for robust RCTs. IBS, irritable bowel syndrome; QOL, quality of life.

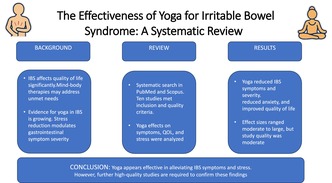

## 1 Introduction

Irritable bowel syndrome (IBS) is a prevalent condition affecting about 5%–10% of the general population, with a significant impact on patients' quality of life (Huang et al. 2023). It is characterized by recurrent abdominal pain and bloating with a predominance of diarrhea and constipation or alternation of these signs (Sebastián Domingo 2022). Different factors contribute to the onset and course of this condition, including genetic predisposition, type of diet, and gut microbiome composition (Black and Ford 2020). Of note, a substantial part of patients shows psychiatric comorbidity and, in particular, affective disorders, thus reflecting the contribution of mind and emotional components in the etiology of IBS (Staudacher et al. 2023). Conventional treatment consists of symptomatic management of diarrhea, constipation, and abdominal pain and the administration of psychotherapy or antidepressants in case of concomitant mental conditions (Camilleri 2021). However, a large part of subjects affected by IBS does not achieve remission after standard management (Card et al. 2014). For this reason, in recent years, complementary medicine has been applied to IBS patients, including a diet low in fermentable oligosaccharides, disaccharides, monosaccharides, and polyols (FODMAP) (Black et al. 2022), physical exercise (Radziszewska et al. 2023), and yoga (Patel and Lacy 2016).

Yoga can be defined as the science of liberation from human suffering, and several data indicate its beneficial role in several chronic medical conditions, including gastrointestinal diseases (Thakur et al. 2024). An unresting mind would not only be associated with psychological symptoms; however, it would also have deleterious effects on the physical body due to unhealthy actions (such as an unregulated diet). Patanjali's classical yoga system involves different types of practices, such as ethical prescriptions, postures (*Asanas*), breath control (*Pranayama*), and meditation (*Dhyana*) (Varambally and Gangadhar 2016). Traditional yoga also includes purificatory procedures (*Shatkriyas*) that are aimed at the health of various organs: cleansing the stomach (*Dhauti*) and isolation/manipulation of abdominal muscles (*Nauli*) represent the techniques targeting the digestive system (Swathi et al. 2021). From a yogic perspective, IBS would be the result of a dysfunction of the third chakra (situated in the upper abdomen) in concomitance with an unresting mind (upper chakras) (Kumar and Sumithra 2022). An ideal practice should, therefore, include seated forward bends that calm the mind and twists that promote activation of the oblique abdominal muscles and massage of the internal organs.

The purpose of the present review is to critically summarize the available data about the effectiveness of yoga in IBS.

## 2 Materials and Methods

The project of the present review was registered on the Open Science Network. A comprehensive literature search was performed in PubMed and Scopus until 31st March 2025. The search focused on studies investigating yoga practices, such as physical postures (asanas), pranayama (breathing exercises), and dhyana (meditation) in the context of IBS. An initial yield of 193 studies was identified, published in English, and conducted up to March 2025.

**FIGURE 1:**
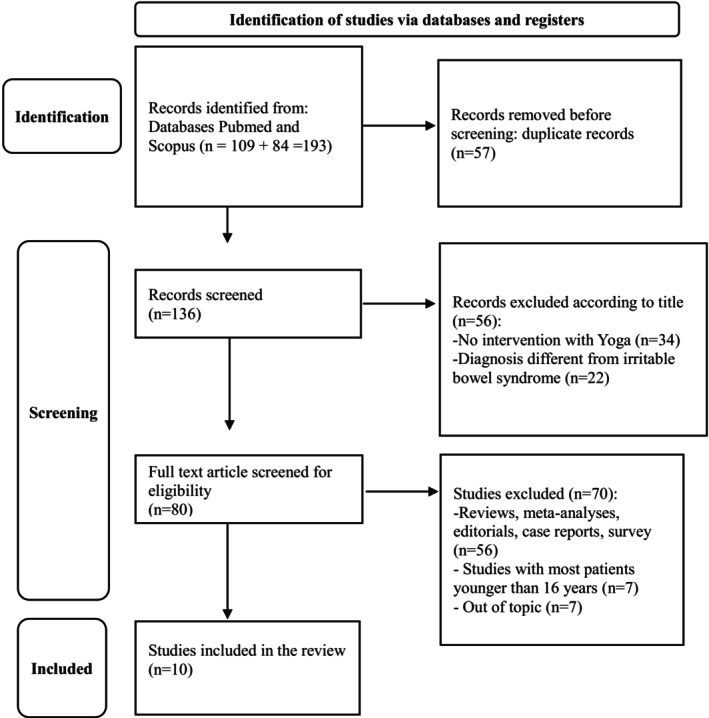
PRISMA diagram for systematic reviews.

Subsequently, we applied the following exclusion criteria: studies with most patients aged < 16 years, review articles, meta‐analyses, systematic reviews, case reports, and case series were all omitted, along with studies focusing on conditions other than IBS and those lacking a yoga‐based intervention (Figure 1).

The methodological quality of these 10 studies was then evaluated according to the Qualitative Assessment Tool for Quantitative Studies (Armijo‐Olivo et al. 2012). Subsequently, we focused on the following aspects of the study: the presence of a controlled design, the tools used, and the yoga outcomes in terms of severity of symptoms and quality of life. Finally, we examined the different yoga modalities across these studies, aiming to propose the most effective approach for managing IBS symptoms.

## 3 Results

Yoga was generally found to be safe, with no serious adverse events attributed to the intervention reported in the included studies. Table 1 summarizes the results of the included studies, while Table 2 reports the results of the quality assessment (none of the included studies was rated as optimal).

**TABLE 1:** Summary of the findings of the included studies.

Study (country)	Yoga type	Sample size	Study duration	Intervention	Assessment tools	Conclusion
Singh et al. (1988)^a^ (India)	Shankha Prakshalana	27	Every 5 days for 4 cycles, then a weekly or bi‐weekly session	Patients ingested warm saline water (1% concentration). After drinking, they performed five specific yoga asanas	Self‐reporting by the patients; no standardized score has been used	85.7% improvement in constipation and 87.4% in vague abdominal pain. 50% improvement in diarrhea and 42.9% in irregular bowel habits. 40% reported increased appetite. 57.1% reduced drug dependency
Taneja et al. (2004) (India)	Surya Nadi Pranayama	22	Twice daily for 2 months	Loperamide (*N* = 12) vs. Yoga (*N* = 9)	TBDQ; STAI; Autonomic Reactivity Tests; EGG; autonomic symptoms score tests; physical flexibility	Yoga and loperamide improved TBDQ and STAI (*p* = 0.001; 0.01 and 0.02; 0.02 resp.), yoga improved autonomic symptoms (*p* = 0.051), and autonomic reactivity tests (*p* = 0.04), Loperamide showed earlier notable improvements in EEG (*p* = 0.007). STAI improved sooner with yoga (*p* = 0.004)
Evans et al. (2014) (USA)	Iyengar Hatha Yoga	51 (30 adolescents 14–17 year) and 21 young adults (18 –26 year)	12 classes over 6 weeks (twice weekly)	Yoga (*n* = 29) vs. wait list control (*n* = 22)	CSI; NRS; GIS; FDI; BSI‐18; FACIT‐Fatigue; PSQI; SF‐36	In young adults, Iyengar Hatha Yoga significantly improved CSI mean change: −2.27 vs. +0.44, *p* = 0.03; GIS *p* = 0.03; BSI‐18, *p* = 0.04; PSQI, *p* = 0.02, FACIT, *p* = 0.05, FDI, *p* = 0.04. Adolescents improved only in SF‐36, *p* = 0.01. In young adults, more in‐person yoga sessions were correlated with greater IBS symptom reduction (*r* = −0.55, *p* = 0.02)
Kavuri et al. (2015) (USA)	Hatha Yoga	78 patients in three groups: 1. Yoga plus minimal treatment. 2. Yoga + unrestricted treatment. 3. Control group	12 weeks thrice a week	Hatha Yoga in group 1 and 2 (total = 51) vs. control group	IBS‐SSS; IBS‐QOL; HADS; IBS‐GAI; Autonomic Symptom Score	Both yoga alone and in combination showed significant improvements in IBS‐SSS (yoga alone: d = 3.60, yoga in combination: *d* = 3.12) and IBS‐QOL versus controls (*p* < 0.001). HADS and IBS‐GAI also improved markedly (*p* < 0.001). Autonomic symptom scores were significantly better in yoga (*p* < 0.01) and combined therapies (*p* < 0.05) by week 12. Both groups reduced medication use (*p* < 0.001) by Week 6, with further reduction at Week 12
Shahabi et al. (2016) (USA)	Iyengar Hatha Yoga	27	16 biweekly sessions (8 weeks). 6 months FU	Yoga (*n* = 17) vs. non—aerobic walking (*n* = 10)	NRS; PANAS‐X; VSI; PHQ‐15; STAI	Yoga and walking both significantly reduced NRS (*p* = 0.001; *p* < 0.01). Walking decreased PANAS‐X (*p* < 0.05), yoga showed stronger benefits on VSI and PHQ −15 (both *p* < 0.05). At 6 months, the walking group maintained or improved GI symptoms (*p* < 0.05), whereas the yoga group showed a partial rebound of symptoms
Schumann et al. (2018) (Germany)	Hatha Yoga	27	12 weeks twice—weekly	Yoga (*n* = 30) vs. low FODMAP diet (*n* = 29)	IBS‐SSS, IBS‐QOL, SF‐36, HADS, CPSS, PSQ, BAQ, BRS	IBSSSS: *p* < 0.001 for both groups at 12w (low FODMAP > yoga: *d* = 0.37) and 24w; IBS‐QOL: low‐FODMAP showing greater food avoidance *p* = 0.005 (12w); SF‐36: no difference between groups; HADS: yoga improved anxiety subscale *p* = 0.025; CPSS: both groups improved (p not specified); PSQ: no difference between groups; BAQ: yoga showing a significant benefit at week 24 *p* = 0.017; BRS: BRS2 *p* = 0.027 (in favor of yoga), BRS1 no difference between groups
Tavakoli et al. (2019) (Iran)	Laughter yoga	60	Seven structured sessions	Three groups (*n* = 20 each): Group A: laughter yoga therapy Group B: Antidepressant (sertraline 50–200 mg/day) Group C: symptomatic treatment (control)	IBS‐SSS, BAI	IBS‐SSS: pre‐intervention (between groups): *p* = 0.83; post‐intervention (between groups): *p* = 0.006; pre‐post intervention difference (within groups): *p* = 0.023 (significant). BAI: Pre‐intervention (between groups): *p* = 0.78; Post‐intervention (between groups): *p* = 0.1 (not significant); Pre‐post intervention difference (within groups): *p* = 0.05 (significant). Improvements on IBS‐SS and BAI were more marked for the yoga group (IBS‐SS scores: yoga versus sertraline: *d* = 0.27; yoga versus symptomatic treatment: *d* = 0.75; sertraline versus symptomatic treatment: *d* = 0.42)
D'Silva et al. (2023) (Canada)	Upa Yoga	63	Eight weekly sessions plus daily home practice	Upa Yoga (*n* = 27) vs. control (*n* = 36)	IBS‐SSS, IBS‐QOL, GAD‐7, PHQ‐9, PSS, COVID‐19 SS, MFIS‐21, PHQ‐15, SCS‐SF	Yoga improved better in: IBS‐QOL *p* = 0.030, MFIS‐21 *p* = 0.035, PSS *p* = 0.040. With regard to IBS‐SSS scores patients treated with yoga ameliorated more than controls (*d* = 0.37)
Chao et al. (2024) (Taiwan)	Hatha yoga	31	6 weeks: thrice‐weekly online yoga sessions	Three groups: (EP) Yoga + probiotics, (EC)Yoga + placebo, (P) probiotics only	IBS‐QOL, BSRS‐5, 16S rRNA, HRR, IPAQ	IBS‐QOL: EP group (−18 points, *p* < 0.001), EC group (−9.7 points, *p* < 0.05), P group (non‐significant); BSRS‐5: EP and EC groups (non‐significant); 3‐min Step Test (HRR): EP group (*p* < 0.01), EC and P groups (non‐significant); 16S rRNA: Klebsiella reduction in EP group (*p* < 0.05), Prevotella reduction in EC group (*p* < 0.05), F/B ratio and alpha diversity (non‐significant)
Kern et al. (2024)^a^ (Sweden)	Ashtanga Yoga	10	10 weeks, 4 times per week	Ashtanga yoga intervention at baseline and after 6 months of follow up	IBS‐SSS, IBS‐QOL, GSRS‐IBS, VSI, HADS, PHQ‐12	After intervention: IBS‐SSS (*p* = 0.004, *d* = 1.38), IBS‐QOL (*p* = 0.000), GSRS‐IBS (*p* = 0.016), VSI (*p* = 0.008), HADS‐Anxiety (*p* = 0.009), HADS‐Depression (*p* = 0.005), PHQ‐12 (*p* = 0.012) After 6 months: IBS‐SSS (0.006), IBS‐QOL (0.002), VSI (0.031), PHQ‐12 (0.009)

Abbreviations: 16S rRNA, 16S Ribosomal RNA Gene Sequencing; BAI, Beck Anxiety Inventory; BAQ, Body Awareness Questionnaire; BRS, Body Responsiveness Scale; BSI‐18, Brief Symptom Inventory‐18; BSRS‐5, Brief Symptom Rating Scale (5‐item); btw, between; CPSS, Cohen Perceived Stress Scale; CSI, Child Somatization Inventory; *d*, Cohen's *d* effect size; EGG, Surface Electrogastrography; FACIT‐Fatigue, Functional Assessment of Chronic Illness Therapy‐Fatigue; F/B, Firmicutes/Bacteroidetes; FDI, Functional Disability Inventory; FODMAP, fermentable oligosaccharides, disaccharides, monosaccharides, and polyols; GIS, Global Improvement Scale; GSRS‐IBS, Gastrointestinal Symptom Rating Scale—Irritable Bowel Syndrome version; HADS, Hospital Anxiety and Depression Scale; HRR, Heart Rate Reserve; IBS‐GAI, Irritable Bowel Syndrome‐Global Assessment of Improvement; IBS‐QOL, Irritable Bowel Syndrome‐Quality of Life; IBS‐SSS, IBS‐Symptom Severity Scoring System; IPAQ, International Physical Activity Questionnaire; MFIS, Modified Fatigue Impact Scale; NRS, Numeric Rating Scale (0–10); PANAS‐X, Positive and Negative Affect Schedule (Positive Affect, Negative Affect); PHQ‐12, Patient Health Questionnaire—12 Item; PHQ‐15, Patient Health Questionnaire‐15 (Somatic Symptoms); PSQ, Perceived Stress Questionnaire; PSQI, Pittsburgh Sleep Quality Index; PSS, Perceived Stress Scale; SF‐36, Short Form‐36 Health Survey; STAI, Spielberger's State–Trait Anxiety Inventory; TBDQ, Talley's Bowel Disease Questionnaire; USA, United States of America; Vs, versus; VSI, Visceral Sensitivity Index; W, week; y, years.

^a^ Non‐controlled study.

**TABLE 2:** Evaluation of the quality of the included studies.

Study	Quality rating
Singh et al. (1988)	3
Taneja et al. (2004)	3
Evans et al. (2014)	2
Kavuri et al. (2015)	2
Schumann et al. (2018)	2
Shahabi et al. (2016)	3
Tavakoli et al. (2019)	2
D'Silva et al. (2023)	2
Kern et al. (2024)	3
Chao et al. (2024)	3

*Note:* Global rating was performed according to these criteria (Qualitative Assessment Tool for Quantitative Studies): (1) Selection Bias (sample size power and number of subjects who agreed to participate into the study). (2) Study Design (randomized versus non‐randomized trials). (3) Confounders (Yes/No). (4) Blinding (Yes/No). (5) Data collection methods (self‐reported data, observations by investigators or medical records). (6) Presence of description of numbers and reasons for withdrawals and drop‐outs. 1 = strong (no weak ratings according to above criteria). 2 = moderate (one weak rating according to above criteria). 3 = weak (two or more weak ratings according to above criteria).

In these studies, yoga intervention programs for patients with IBS were either designed as simple feasibility pilot studies (Singh et al. 1988; Kern et al. 2024) or compared to walking (Shahabi et al. 2016) standard pharmacological (Taneja et al. 2004; Tavakoli et al. 2019) or dietary therapy (Schumann et al. 2018), probiotics (Chao et al. 2024), or non‐intervention control groups (Kavuri et al. 2015; D'Silva et al. 2023; Evans et al. 2014).

A variety of yoga styles were tested, including Iyengar‐based hatha yoga (Kavuri et al. 2015; Shahabi et al. 2016; Chao et al. 2024; Evans et al. 2014) surya nadi pranayama (Taneja et al. 2004), shankha prakshalana (Singh et al. 1988), upa yoga (D'Silva et al. 2023), ashtanga yoga (Kern et al. 2024) and laughter yoga (Tavakoli et al. 2019).

Program durations ranged 6–12 weeks. Sample sizes per study varied from very small feasibility cohorts (*N* = 10) (Kern et al. 2024) to larger randomized trials (*N* = 78) (Kavuri et al. 2015).

### 3.1 IBS Symptom Severity

Half of the studies used the changes on the IBS Symptom Severity Scale (IBS‐SSS) as the primary outcome (Kavuri et al. 2015; Schumann et al. 2018; Tavakoli et al. 2019; D'Silva et al. 2023; Kern et al. 2024) An 8‐week online Upa yoga trial found that IBS‐SSS decreased significantly in the yoga group (mean change ≈ −54.7, *p* = 0.028) with no significant change in the advice‐only control group (*p* = 0.277) (D'Silva et al. 2023). In this study, 37% of yoga participants achieved a ≥ 50 point IBS‐SSS score reduction compared to 20% of controls, although this difference was not statistically significant (*p* = 0.242). A 10‐week Ashtanga yoga feasibility study (Kern et al. 2024) reported a large drop in IBS‐SSS scores immediately post‐intervention (*p* = 0.004) and sustained improvement at 6‐month follow‐up (*p* = 0.006). In contrast, although the Gastrointestinal Symptom Rating Scale IBS version (GSRS‐IBS) score showed a significant reduction immediately post‐intervention (*p* = 0.016), this effect was not maintained at 6 months (*p* = 0.292). A 12‐week intensive yoga program (three sessions/week) documented a 120‐point mean IBS‐SSS reduction in the two yoga arms composed of the yoga plus minimal‐IBS standard treatment arm and yoga with unrestricted‐IBS standard treatment (combination) (Kavuri et al. 2015). By week 6, over 90% of patients in these two groups reached the clinically meaningful ≥ 50‐point drop in IBS‐SSS, progressing to nearly 100% at Week 12. Similarly, the amelioration of the IBS Global Assessment of Improvement (IBS‐GAI) score was larger in both yoga groups over the control (*p* < 0.001). No statistically significant differences emerged between the yoga group and combination one in symptom control (Kavuri et al. 2015). Notably, in a head‐to‐head trial comparing 12 weeks of yoga (twice weekly sessions) to a low‐FODMAP diet, both groups showed significant within‐group IBS‐SSS improvement at week 12 (*p* < 0.001 for both groups) and maintained these values at week 24 (*p* < 0.001) (Schumann et al. 2018). No significant difference was found between yoga and FODMAP diet groups in IBS‐SSS change at 12 weeks (mean difference ~32 points, *p* = 0.151) or 24 weeks (*p* = 0.081). In a randomized controlled study, the effects of laughter yoga on patients with IBS were compared to sertraline and control (Tavakoli et al. 2019). Symptom severity was assessed before and after a 7‐week intervention period. The study demonstrated a statistically significant reduction in IBS symptom severity measured by IBS‐SSS after intervention, particularly in the laughter yoga group (*p* = 0.006).

Other studies have assessed the effects of yoga on IBS symptoms using different evaluation tools compared to those employed in the studies mentioned above (Singh et al. 1988; Taneja et al. 2004; Shahabi et al. 2016; Evans et al. 2014).

Shankha Prakshalana represents one of the earliest attempts to explore the therapeutic role of yogic practices in managing IBS (Singh et al. 1988). In this study, 27 IBS patients reported significant symptom improvement following Shankha Prakshalana, with reductions in constipation (85.7%) and abdominal pain (87.4%), though no *p*‐values or statistical analyses were provided. In a small pilot trial of 16 biweekly sessions (8 weeks in total) comparing Iyengar‐based hatha yoga to a walking‐based exercise as a control, no significant differences in IBS symptom improvement measured by the Numeric Rating Scale (NRS) were found (Shahabi et al. 2016). However, the pre‐post symptom improvement in the yoga group was statistically significant (*p* < 0.05). A study compared the effects of surya nadi pranayama and loperamide on symptom reduction in patients with diarrhea‐predominant irritable bowel syndrome (IBS‐D) (Taneja et al. 2004). The yogic intervention and loperamide treatment significantly decreased bowel symptom scores assessed by Talley's Bowel Disease Questionnaire (TBDQ) over 2 months. The improvement was more significant in the yoga group (*p* = 0.001) than in the loperamide group (*p* = 0.01). In a randomized study, a 6‐week Iyengar Yoga intervention was compared to a waitlist control in adolescents (14–17 years) and young adults (YA, 18–26 years) (Evans et al. 2014). IBS symptoms were assessed using the Child Somatization Inventory (CSI), and abdominal pain was assessed with the NRS. Unlike adolescents, YA showed a significant reduction in CSI score (mean change = −2.27 vs. +0.44; *p* = 0.03). Although NRS pain scores were not statistically different, 46% of YA and 44% of adolescents reported clinically meaningful pain reduction. Improvements in YA were sustained at the 2‐month follow‐up. Chao and colleagues did not employ specific gastrointestinal symptom severity tools such as the IBS‐SSS. Instead, they focused on indirect outcomes, primarily assessing quality of life through the IBS‐QOL, reflecting the broader impact of interventions on daily functioning in IBS patients (Chao et al. 2024).

### 3.2 Quality of Life, Physical Function, Anxiety, Depression, and Stress

Several reviewed studies investigated gastrointestinal symptom relief in patients with IBS and addressed broader dimensions of patients' well‐being. These included quality of life, visceral sensitivity, somatic distress, and psychological functioning. To capture these multidimensional outcomes, the trials employed a range of validated assessment tools, such as the Irritable Bowel Syndrome–Quality of Life questionnaire (IBS‐QOL), the 36‐Item Short Form Survey (SF‐36), the Visceral Sensitivity Index (VSI), the 12‐item Patient Health Questionnaire (PHQ‐12), the Hospital Anxiety and Depression Scale (HADS), the State–Trait Anxiety Inventory (STAI), the Perceived Stress Scale (PSS), and the 5‐item Brief Symptom Rating Scale (BSRS‐5) (For more details, refer to Table 1).

In a randomized controlled trial by Taneja and colleagues, 2 months of Surya Nadi Pranayama yoga practice in patients with diarrhea‐predominant IBS resulted in a significant reduction in state anxiety (*p* = 0.004), comparable to that obtained by the group in treatment with loperamide. The yoga group also benefited from a significant improvement in autonomic symptoms (*p* = 0.05) and parasympathetic reactivity (*p* = 0.04). Gastric motility improved in both groups, with a more marked effect at 1 month in the loperamide group (*p* = 0.007) (Taneja et al. 2004). Evans et al. showed that YA in the Iyengar yoga group exhibited significant improvements in psychological distress, reduced functional disability, and improved sleep quality and fatigue levels compared to the wait‐list group (Evans et al. 2014). In another study testing the effectiveness of 16‐session Iyengar‐based practice, the yoga group showed significant pre‐post reductions in visceral sensitivity and somatic symptom severity (both *p* < 0.05) compared to a walking program in IBS patients (Shahabi et al. 2016). In a 12‐week randomized trial of a comprehensive hatha yoga module for IBS conducted by Kavuri et al. participants in both the yoga and yoga‐plus‐medication groups demonstrated significantly greater improvements in IBS‐QOL compared to a wait‐list control (*p* < 0.001). These groups also showed significant reductions in anxiety and depression scores. Medication use decreased significantly by week 6 (*p* < 0.001) and remained low until week 12 (Kavuri et al. 2015). Schumann et al. compared 12 weeks of hatha yoga to a low‐FODMAP diet in IBS. Both groups achieved significant within‐group reductions in perceived stress measures over the treatment period. There were no overall between‐group differences in health‐related quality of life (SF‐36 or total IBS‐QOL); however, diet‐group patients reported a specific decline in one QOL domain related to food avoidance (IBS‐QOL subscale, *p* = 0.005). The yoga group showed a greater improvement in physical health status and had significantly lower anxiety on HADS score than the diet group at 12 weeks (HADS‐Anxiety *p* = 0.025). HADS depression scores were similar between groups (Schumann et al. 2018). Tavakoli et al. compared laughter yoga, antidepressant medication (sertraline), and standard care in an IBS randomized trial. All groups showed decreased anxiety severity after the intervention, with the most significant reduction observed in the laughter yoga arm. However, the between‐group difference was insignificant (*p* = 0.1) (Tavakoli et al. 2019). D'Silva et al. conducted an 8‐week virtual upa yoga trial. The yoga group showed significantly higher post‐intervention IBS‐QOL (*p* = 0.030), lower fatigue (*p* = 0.035), and perceived stress levels (*p* = 0.040) compared to an education‐only control. By contrast, there were no significant differences between groups in general anxiety (GAD‐7) or depression (PHQ‐9) (D'Silva et al. 2023). Kern et al. conducted a feasibility study of an Ashtanga yoga group program in a primary care setting. Following the 10‐week intervention, participants showed significant improvements in IBS‐QOL scores, increasing from 64.5 to 72.3 (*p* = 0.004), a significant reduction in visceral sensitivity measured by the VSI score from 38.8 to 25.9 (*p* = 0.005), and a decrease in somatic symptom burden (PHQ‐12 scores from 10.0 to 6.9, *p* = 0.003). Scores for anxiety and depression, assessed by the HADS, also declined after the intervention (*p* = 0.014; *p* = 0.034). Most improvements were sustained at the 3‐month follow‐up. Reductions in general anxiety and depression were no longer statistically significant at 6 months (Kern et al. 2024). Finally, the group by Chao and collaborators evaluated a 6‐week intervention of a hatha yoga‐based online program plus probiotics versus yoga plus placebo and versus probiotics‐only. The combined yoga–probiotic group and yoga plus placebo significantly ameliorated IBS‐QOL scores (*p* < 0.001 and *p* < 0.05, respectively). Psychological distress levels (BSRS‐5 score) tended to decrease in the yoga groups, although this reduction did not reach statistical significance (Chao et al. 2024).

### 3.3 Type of Practice

Half of the studies used hatha yoga (Kavuri et al. 2015; Schumann et al. 2018; Chao et al. 2024), some of them with a program based on Iyengar's teachings (Shahabi et al. 2016; Evans et al. 2014). Singh and collaborators (Singh et al. 1988) recurred to shanka (conch shell) prakshalana (cleaning), a hatha‐yoga‐based technique of intestine purification that was detailed in the textbook Gheranda Samhita (Mishra and Dash 2017). It consists of quickly drinking two glasses of a warm saline solution and performing positions composed of stretches, lateral flexions, and twists of the spine. Taneja and co‐authors (Taneja et al. 2004) tested a type of yogic practice that combined right‐nostril breathing (energizing pranayama) with a set of 12 poses (mainly backbends and forward bends). The practice proposed by Evans and co‐authors in the article includes several inversions (e.g., shoulder stand) and reclined positions (e.g., Supta Buddha Konasana—reclining fixed angle posture). Kavuri et al. (2015) proposed a practice that includes a mix of postures, breathing techniques, and purifications. Shahabi and co‐authors (Shahabi et al. 2016) administered a sequence consisting of seated poses, inversions, backbends, twists, and restorative supine poses. Schumann and collaborators (Schumann et al. 2018) applied a complex yoga method that included one weekly class with yoga postures and pranayama and a second weekly class of mantra meditation and yoga nidra (deep relaxation). The postures administered in each class depended on the previous ones, with the difficulty and intensity levels being increased carefully as the program progressed; no specific class of poses was privileged (Schumann et al. 2018). Laughter yoga was tested by Tavakoli et al. (2019) (Tavakoli et al. 2019). It is a method developed by the Indian physician Kataria on the basis that artificial and natural laughter have a similar effect on the body (Tabei et al. 2025). Other researchers (D'Silva et al. 2023) applied upa yoga, a variant of hatha yoga. The Isha Foundation of Inner Sciences developed it. It consists of: (1) arm movements and neck rotations as a muscle warm‐up, (2) asanas that include sitting and squatting poses, (3) breathing practices or alternate nostril breathing (*Nadi‐Shodhana*), (4) mantra meditation consisting of OM chanting, and (5) meditation with breath watching (Kumar and Sumithra 2022). Chao and collaborators (Chao et al. 2024) tested a practice consisting mainly of sitting or lying positions with flexion of the femurs on the hips. Finally, modifications of the Ashtanga yoga primary series with a focus on forward bends were administered by Kern and co‐authors (Kern et al. 2024).

### 3.4 Home Practice Adherence

Adherence to home‐based yoga protocols varied significantly across studies, possibly influencing outcomes. In the Iyengar yoga pilot study by Shahabi and collaborators (Shahabi et al. 2016) only 45% of participants continued weekly practice 6 months' post‐intervention, compared to 80% in the walking group (*p* < 0.05). This decline may account for symptom recurrence in the yoga group, emphasizing the importance of long‐term adherence. In contrast, structured home programs showed better compliance. In the Ashtanga feasibility trial (Kern et al. 2024), participants were instructed to practice 45 min at home at least 4 days per week, achieving an average of three sessions per week and high‐class attendance (8/9 sessions). Higher adherence was linked to more significant and clinically meaningful reductions in IBS symptoms (D'Silva et al. 2023). Furthermore, age differences affected adherence and outcomes. In YA, a moderate inverse correlation (*r* = −0.55, *p* = 0.02) was found between the number of in‐person yoga hours and IBS symptom severity, suggesting greater exposure yields greater benefits (Evans et al. 2014).

## 4 Discussion

Originating in ancient India, yoga is now mainstream in Western health culture and is frequently used to promote general wellness and stress reduction. There is growing evidence that yoga produces measurable benefits in various chronic conditions. For example, randomized trials showed that yoga can relieve chronic lower back pain and improve functional ability (Anheyer et al. 2022). Meta‐analyses in mental health indicate that regular yoga practice can reduce symptoms of depression and anxiety (Wu et al. 2023). Yoga also positively affected cardiovascular risk factors, arthritis symptoms, and overall quality of life in different patient populations, highlighting its broad therapeutic potential (Cramer et al. 2014; Kan et al. 2016; Bartlett et al. 2013). Given these benefits, it is not surprising that many IBS patients are turning to yoga as a coping strategy for their gastrointestinal condition. Across most IBS studies, yoga interventions led to reduced abdominal pain and bloating, improved bowel habits, decreased anxiety and depression scores, and enhanced quality of life relative to usual care or wait‐list controls. Of note, the studies that fixed the change in IBS‐SSS score as the primary outcome showed a moderate–large effect size of yoga on the amelioration of symptoms compared to the controls (Cohen's *d* range: 0.37–3.60). These preliminary findings suggest that yoga could favorably influence both the physical and psychological aspects of IBS, aligning with the multifactorial needs of this patient group. In a 2023 mixed‐methods survey of 219 IBS patients, 32% reported current yoga practice (Doyle and Cartwright 2023). Yoga incorporates gentle physical activity, which can improve abdominal muscle tone and bowel motility, along with breathing and mindfulness components that downregulate the sympathetic nervous system and attenuate the stress response (Josefsson et al. 2019).

Despite these promising indications, the current literature on yoga for IBS is still at the very beginning and has notable limitations. Furthermore, all the available studies show one or more weaknesses. Most published trials have small sample sizes and are often feasibility or pilot studies. A related issue is the heterogeneity of yoga interventions and the outcome measures used. There is no universally accepted “yoga protocol” for IBS; available studies vary primarily in the applied yoga style. Yoga interventions differ significantly in quality and intensity of postures, duration, inclusion of meditation, and population studied. This makes direct comparisons difficult and prevents meta‐analytic synthesis. Few studies dissect the active components of these multifactorial interventions. For instance, it is unclear whether yoga benefits derive more from its physical (asana), breathing (pranayama), or meditative (dhyana) aspects (Hamal et al. 2025). Some patients may benefit most from gentle restorative yoga focused on stress reduction, while others might need more vigorous practices to aid constipation. Treatment durations in trials range from a few weeks to several months, making it difficult to determine the optimal dose of yoga for sustained IBS relief. The studies used different IBS symptom severity scales, quality‐of‐life questionnaires, and psychological inventories, with poor standardization.

Additionally, some investigations combined mixed patient populations. For example, including both pediatric and adult IBS patients or patients with IBS alongside other functional disorders potentially obscured treatment effects in the specific adult IBS subgroup of interest. There are few “head‐to‐head” studies with precise therapy (Taneja et al. 2004; Tavakoli et al. 2019). Most other published studies tend to compare yoga with non‐pharmacological control groups or “standard” treatments, typically consisting of dietary advice, psychotherapy, or generic medications. Although gastroenterologists generally acknowledge yoga as a safe practice with stress‐reducing and well‐being benefits, its incorporation into routine therapeutic recommendations remains uncommon (D'Silva et al. 2023). This is mainly due to two key barriers: insufficient high‐quality, condition‐specific evidence demonstrating its efficacy for gastrointestinal outcomes and limited knowledge regarding referral pathways and appropriate program prescription. Many clinicians report feeling unqualified or uncertain about effectively guiding patients toward credible, structured yoga options. Several critical developments are required to advance yoga as a viable therapeutic modality within gastroenterology. These include rigorous clinical trials that establish its effectiveness specifically for gastrointestinal disorders, creating standardized and evidence‐based protocols suitable for medical recommendation, targeted educational initiatives to enhance physician knowledge and confidence, and clear, accessible referral systems connecting patients with qualified instructors or validated digital platforms. These measures would help close the gap between positive professional attitudes and actual clinical implementation. There is a clear need for more rigorous and standardized research to definitively establish the role of yoga in managing IBS. Experts have begun calling for the adoption of common frameworks and protocols in this field (Ward et al. 2022). Future trials should measure potential mediators (e.g., stress hormones or autonomic parameters) multiple times to test causality rather than simple associations.

This review expands the scope of yoga for IBS by covering diverse practices, from asana and pranayama to meditation, laughter yoga, and upa yoga. It compares yoga with both pharmacological and non‐pharmacological interventions and considers both gastrointestinal and psychological outcomes, reflecting the biopsychosocial paradigm of IBS pathophysiology. By summarizing intervention styles, duration, and adherence, it also provides a practical basis for developing future standardized protocols.

## Conflicts of Interest

The authors declare no conflicts of interest.

## Data Availability

The data that support the findings of this study are available from the corresponding author upon reasonable request.
